# Post-operative Atrial Fibrillation Impacts on Outcomes in Transcatheter and Surgical Aortic Valve Replacement

**DOI:** 10.3389/fcvm.2021.789548

**Published:** 2021-11-29

**Authors:** Hyung Ki Jeong, Namsik Yoon, Ju Han Kim, Nuri Lee, Dae Yong Hyun, Min Chul Kim, Ki Hong Lee, Yo Cheon Jeong, In Seok Jeong, Hyun Ju Yoon, Kye Hun Kim, Hyung Wook Park, Youngkeun Ahn, Myung Ho Jeong, Jeong Gwan Cho

**Affiliations:** ^1^Division of Cardiology, Department of Internal Medicine, School of Medicine, Wonkwang University, Iksan, South Korea; ^2^Division of Cardiology, Department of Internal Medicine, Chonnam National University Medical School, Gwangju, South Korea; ^3^Department of Thoracic and Cardiovascular Surgery, Chonnam National University Medical School, Gwangju, South Korea

**Keywords:** atrial fibrillation, incidence, aortic stenosis, transcatheter aortic valve replacement, surgical aortic valve replacement, outcome

## Abstract

**Background:** Atrial fibrillation (AF) in severe aortic stenosis (AS) has poor outcomes after transcatheter and surgical aortic valve replacement (TAVR and SAVR, respectively). We compared the incidence of AF after aortic valve replacement (AVR) according to the treatment method and the impact of AF on outcomes.

**Methods:** We investigated the incidence of AF and clinical outcomes of AVR according to whether AF occurred after TAVR and SAVR after propensity score (PS)-matching for 1 year follow-up. Clinical outcomes were defined as death, stroke, and admission due to heart failure. The composite outcome comprised death, stroke, and admission due to heart failure.

**Results:** A total of 221 patients with severe AS were enrolled consecutively, 100 of whom underwent TAVR and 121 underwent SAVR. The incidence of newly detected AF was significantly higher in the SAVR group before PS-matching (6.0 vs. 40.5%, *P* < 0.001) and after PS-matching (7.5 vs. 35.6%, *P* = 0.001). TAVR and SAVR showed no significant differences in outcomes except in terms of stroke. In the TAVR group, AF history did not affect the outcomes; however, in the SAVR group, AF history affected death (log rank *P* = 0.038). Post-AVR AF had a worse impact on admission due to heart failure (log rank *P* = 0.049) and composite outcomes in the SAVR group. Post-AVR AF had a worse impact on admission due to heart failure (log rank *P* = 0.008) and composite outcome in the TAVR group.

**Conclusion:** Post-AVR AF could be considered as a predictor of the outcomes of AVR. TAVR might be a favorable treatment option for patients with severe symptomatic AS who are at high-risk for AF development or who have a history of AF because the occurrence of AF was more frequent in the SAVR group.

## Introduction

For decades, surgical aortic valve replacement (SAVR) has been the definitive treatment option for patients with severe symptomatic aortic valve stenosis (AS). However, patients with severe AS tend to be older, frail, and usually have multiple comorbidities. Therefore, a substantial proportion of AS patients are ineligible for SAVR. Transcatheter aortic valve replacement (TAVR) has emerged as a treatment option for severe symptomatic AS (1–4) and has taken the place of SAVR for some patients who are at high- or intermediate-risk for SAVR (1, 4). Recent data have also shown that TAVR is safe and effective for low-risk patients with SAVR (2, 3). Therefore, TAVR is now performed worldwide and is favored by some physicians because of the short duration of hospitalization and low periprocedural morbidity. With the progression of technology and learning curves, the number of TAVR procedures has also increased in Korea (5, 6).

Atrial fibrillation (AF) is the most common arrhythmia that requires appropriate management, such as rhythm control, rate control, and anticoagulation. AF is known to be associated with adverse clinical outcomes, such as heart failure, systemic embolism, stroke, or death. Because many cases of severe AS and AF are degenerative, their incidence is high in old patients, and they share similar risk factors contributing to worse clinical outcomes. Moreover, it is well-known that AF itself is an independent predictor of long-term mortality and heart failure in patients with AS (7). Previous studies have demonstrated that postoperative AF or pre-existing AF is associated with poor prognosis after TAVR (8, 9). Postoperative AF is also known to have a detrimental effect after cardiac surgery (10, 11). However, there are only a few studies comparing effects of AF on the outcomes of TAVR vs. SAVR.

Therefore, we aimed to compare the AF status in TAVR and SAVR, and its impact on the development of admission due to heart failure, death, stroke. Moreover, we compared the composite outcomes according to the management method for severe AS.

## Methods

### Study Population

A total of 221 patients with severe symptomatic AS were consecutively enrolled from January 2016 to December 2019 at the Department of Cardiology and Thoracic and Cardiovascular Surgery at Chonnam National University Hospital in Gwangju, South Korea. Among them, 100 patients underwent TAVR and 121 underwent SAVR.

We retrospectively reviewed the medical records and analyzed the clinical data of all patients before and during the regular follow-up period. The data included previous medical history, echocardiographic parameters, laboratory results, and clinical outcomes. We analyzed the AF status and clinical outcomes according to the occurrence of AF in each aortic valve replacement (AVR) method. We performed propensity score (PS)-matching to homogenize the group characteristics. After PS-matching, the patient groups were matched 1:1 (TAVR, *n* = 53; SAVR, *n* = 53). The follow-up period was 1 year or until the first occurrence of any study outcome since enrollment.

The study was approved by the Ethics Committee of Chonnam National University Hospital, Gwangju, South Korea (IRB No., CNUH-2021-022). The requirement for informed consent was waived because the study was a retrospective analysis.

### TAVR Procedure

Eligibility for TAVR was discussed by a multidisciplinary team, including cardiologists, cardiac surgeons, and anesthesiologists. The team decided to perform the procedure after reaching a consensus. All patients who underwent TAVR received either the balloon-expandable Edwards SAPIEN 3 transcatheter heart valves (Edwards LifeSciences, Irvine, CA, USA) or the self-expandable Medtronic Evolut R or Evolut PRO (Medtronic, Minneapolis, MN, USA). The approach route was mostly via the transfemoral (*n* = 99), except in one case, in which it was via the subclavian artery. The patient who was approached via the subclavian artery had very small, tortuous right and left superficial femoral arteries on computer tomographic angiography image, which would have made it difficult to insert the guiding catheters. All patients received aspirin (300 mg) and clopidogrel (300 mg) as loading doses before the TAVR procedure. Anticoagulation with unfractionated heparin was maintained during the procedure by monitoring the activation clotting time. Dual antiplatelet therapy (aspirin, 100 mg and clopidogrel, 75 mg) was maintained for at least 3 months after the procedure depending on the patient's clinical condition.

### Definition and Outcomes

Severe AS was defined as: effective orifice area of the aortic valve ≤ 1.0 cm^2^, effective orifice area index ≤ 0.8 cm^2^/m^2^, mean pressure gradient ≥40 mmHg, and/or jet velocity ≥4.0 m/s by transthoracic echocardiography examination.

Post-AVR AF was defined as the occurrence of AF, irrespective of whether the patients had a history of AF post-operatively. Newly detected AF was defined as the documentation of AF during admission following the procedure without a history of AF.

The clinical outcome was assessed by death, stroke, admission due to heart failure, and implantation of a permanent pacemaker. The composite outcome was defined as the composition of death, stroke, and admission due to heart failure.

### Statistical Analyses

Statistical analyses were performed using SPSS version 25.0 for Windows (SPSS, Inc., Chicago, Illinois, USA). Continuous variables were presented as the mean values ± standard deviations. Student's *t*-test was used to evaluate the differences between continuous variables. Categorical variables were presented as percentages and frequencies, and were analyzed using the chi-square test or Fisher's exact test, as appropriate. PS-matching was performed to obtain similar baseline characteristics for each group. The PS was calculated using multivariable logistic regression incorporating frequently used variables and potential risk factors, including age, sex, hypertension, diabetes mellitus, chronic kidney disease, history of stroke or transient ischemic attack, previous myocardial infarction, or heart failure. Matching was performed using a greedy matching protocol (1:1 nearest neighbor matching without replacement). The 1 year survival rate was estimated using the Kaplan–Meier method, and the curves were compared using the log-rank test. Comparison of clinical outcomes was adjusted using Cox proportional hazards model. In all statistical tests, a two-sided *P* < 0.05 was considered statistically significant.

## Results

### Baseline Characteristics

The baseline characteristics of the patients are shown in Table 1. Before PS-matching, 100 patients underwent TAVR and 121 patients underwent SAVR. There were no significant differences in sex, height, body weight, or body mass index between the groups. The mean age was higher in the TAVR group than in the SAVR group (79.69 ± 6.1 vs. 68.25 ± 10.3; *P*< *0.001*), and the proportion of patients >65 years was higher in the TAVR group (98.0% vs. 66.9%; *P* < 0.001). The incidence of comorbidities, such as of diabetes mellitus, heart failure, stroke, and chronic kidney disease, was comparable between the two groups. Hypertension and previous myocardial infarctions were more common in the TAVR group. The Society of Thoracic Surgeons (STS) score was similar between the two groups. There was no significant difference in echocardiographic parameters, such as ejection fraction and left atrium size, between the two groups. The CHA2DS2-VASc score was higher in the TAVR group than the SAVR group (4.20 ± 1.4 vs. 2.62 ± 1.6; *P* < 0.001). After PS-matching, the baseline characteristics were comparable between the two groups; there were no significant differences in sex, age, comorbidities, STS risk score, echocardiographic parameters, laboratory data, and CHA2DS2-VASc score.

**Table 1:** Baseline characteristics before and after propensity score matching.

	**Before propensity score matching**			**After propensity score matching**		
	**TAVR (*n* = 100)**	**SAVR (*n* = 121)**	***P*-value**	**TAVR (*n* = 53)**	**SAVR (*n* = 53)**	***P*-value**
Female sex, *n*(%)	57 (57.0)	54 (44.6)	0.067	25 (57.2)	24 (45.3)	0.846
Age, years	79.69 ± 6.1	68.25 ± 10.3	<0.001	77.50 ± 7.0	75.32 ± 5.8	0.083
>65 years	98 (98.0)	81 (66.9)	<0.001	51 (96.2)	50 (94.3)	0.647
Height (cm)	157.40 ± 9.3	159.29 ± 7.7	0.110	159.18 ± 9.4	158.51 ± 8.2	0.706
Weight (kg)	62.95 ± 14.8	60.65 ± 9.8	0.174	62.88 ± 14.6	58.34 ± 9.4	0.073
BMI (kg/m^2^)	1.65 ± 0.2	1.63 ± 0.2	0.451	1.66 ± 0.2	1.60 ± 0.2	0.092
Medical history, *n* (%)
Hypertension	77 (77.0)	66 (54.5)	0.001	39 (73.6)	31 (58.5)	0.151
Diabetes mellitus	30 (30.0)	33 (27.3)	0.655	20 (37.7)	15 (28.3)	0.409
Previous myocardial infarction	17 (17.0)	5 (4.1)	0.001	8 (15.1)	4 (7.5)	0.359
Previous heart failure	22 (22.0)	15 (12.4)	0.057	10 (18.9)	6 (11.3)	0.416
Previous CVA	14 (14.0)	14 (11.6)	0.589	11 (20.8)	8 (15.1)	0.613
CKD or ESRD	11 (11.0)	16 (13.2)	0.615	6 (11.3)	7 (13.2)	0.767
Smoking	20 (20.0)	33 (27.3)	0.208	13 (24.5)	15 (28.3)	0.826
STS score	8.10 ± 0.2	7.93 ± 0.5	0.201	7.84 ± 0.8	7.19 ± 0.9	0.105
Echocardiographic parameter
Ejection fraction (%)	60.53 ± 12.6	61.43 ± 12.6	0.600	60.50 ± 12.7	60.86 ± 13.7	0.888
Mean pressure gradient (mmHg)	50.06 ± 64.6	54.49 ± 18.9	0.466	49.17 ± 14.0	52.76 ±19.8	0.290
AoV area (mm^2^)	0.77 ± 0.4	0.87 ± 0.5	0.120	0.80 ± 0.2	0.83 ± 0.2	0.418
AoV velocity (m/s)	4.68 ± 0.6	4.96 ± 0.6	0.438	4.55 ± 0.6	4.57 ± 0.8	0.856
LA size (mm)	47.32 ± 5.9	48.00 ± 7.3	0.516	47.22 ± 5.7	48.40 ± 7.0	0.416
Laboratory data
Cr	1.10 ± 1.4	1.21 ± 1.0	0.495	1.31 ±1.9	1.18 ± 0.8	0.654
ProBNP	3,391.79 ± 5227.3	3,283.34 ± 4745.6	0.909	4,165.02 ± 6895.8	4,736.16 ± 5853.5	0.742
CHA2DS2-VASc	4.20 ± 1.4	2.62 ± 1.6	<0.001	4.11 ± 1.7	3.43 ± 1.5	0.080

*TAVR, Transcatheter aortic valve implantation; SAVR, Superficial aortic valve replacement; BMI, Body mass index; AoV, Aortic valve; CVA, Cerebrovascular accident; CKD, Chronic kidney disease; ESRD, End-stage renal disease; STS, Society of Thoracic Surgeons; LA, Left atrium*.

### AF Occurrence

Before PS-matching, the history of AF frequency was similar between the two groups. Post-AVR AF was more frequent in the SAVR group than in the TAVR group (55.4 vs. 23.0%; *P* = 0.001). Among the post-AVR AF, the number of newly detected cases of AF was 6 and 49, respectively (TAVR vs. SAVR, 6.0 vs. 40.5%; *P* < 0.001).

After PS-matching, the rates of previous AF were not significantly different between the two groups. Post-AVR AF was higher in the SAVR group than in TAVR group (60.4 vs. 22.6%; *P* < 0.001). The incidence of newly detected AF was also higher in the SAVR group than in the TAVR group (35.6 vs. 7.5%; *P* = 0.001). Additionally, newly detected AF was terminated relatively early; most of the AF was terminated within 1 month, especially in the SAVR group (Table 2).

**Table 2:** Atrial fibrillation occurrence before and after procedure.

	**Before propensity score matching**			**After propensity score matching**		
	**TAVR (*n* = 100)**	**SAVR (*n* = 121)**	***P*-value**	**TAVR (*n* = 53)**	**SAVR (*n* = 53)**	***P*-value**
Past history of AF (%)	24 (24.0)	25 (20.7)	0.552	11 (20.7)	16 (30.2)	0.176
Paroxysmal	12 (12.0)	16 (13.2)	0.322	5 (9.4)	10 (18.9)	0.150
Persistent	12 (12.0)	9 (7.4)	0.322	6 (11.3)	6 (11.3)	1.000
Post-AVR AF (%)	23 (23.0)	67 (55.4)	0.001	12 (22.6)	32 (60.4)	<0.001
Newly detected	6 (6.0)	49 (40.5)	<0.001	4 (7.5)	19 (35.6)	0.001
Termination <1 month	3	45	0.022	3	17	0.001
Termination 1–3 months	2	0	0.010	1	0	1.000
Persistence >3 months	1	4	0.452	0	2	0.495
Known-paroxysmal	5 (5.0)	10 (8.3)	0.337	2 (3.8)	8 (15.1)	0.093
Known-persistent	12 (12.0)	7 (5.8)	0.101	6 (11.3)	5 (9.4)	0.750
Rhythm control
Amiodarone	9 (9.0)	16 (14.9)	0.219	4 (7.5)	9 (17.0)	0.236
DC cardioversion	1 (1.0)	7 (5.8)	0.075	1 (1.9)	6 (11.3)	0.051
Anticoagulation	14 (14.0)	27 (22.3)	0.122	7 (13.2)	11 (20.8)	0.301
Warfarin	3 (3.0)	23 (19.0)	<0.001	1 (1.9)	7 (13.2)	0.060
NOACs	11(11.0)	4 (3.3)	0.023	6 (11.3)	4 (7.5)	0.371

*TAVR, Transcatheter aortic valve implantation; SAVR, Superficial aortic valve replacement; AF, Atrial fibrillation; AVR, Aortic valve replacement; NOACs, Novel oral anticoagulants*.

### Clinical Outcomes

#### Adverse Event Rate: TAVR vs. SAVR

**Figure 1:**
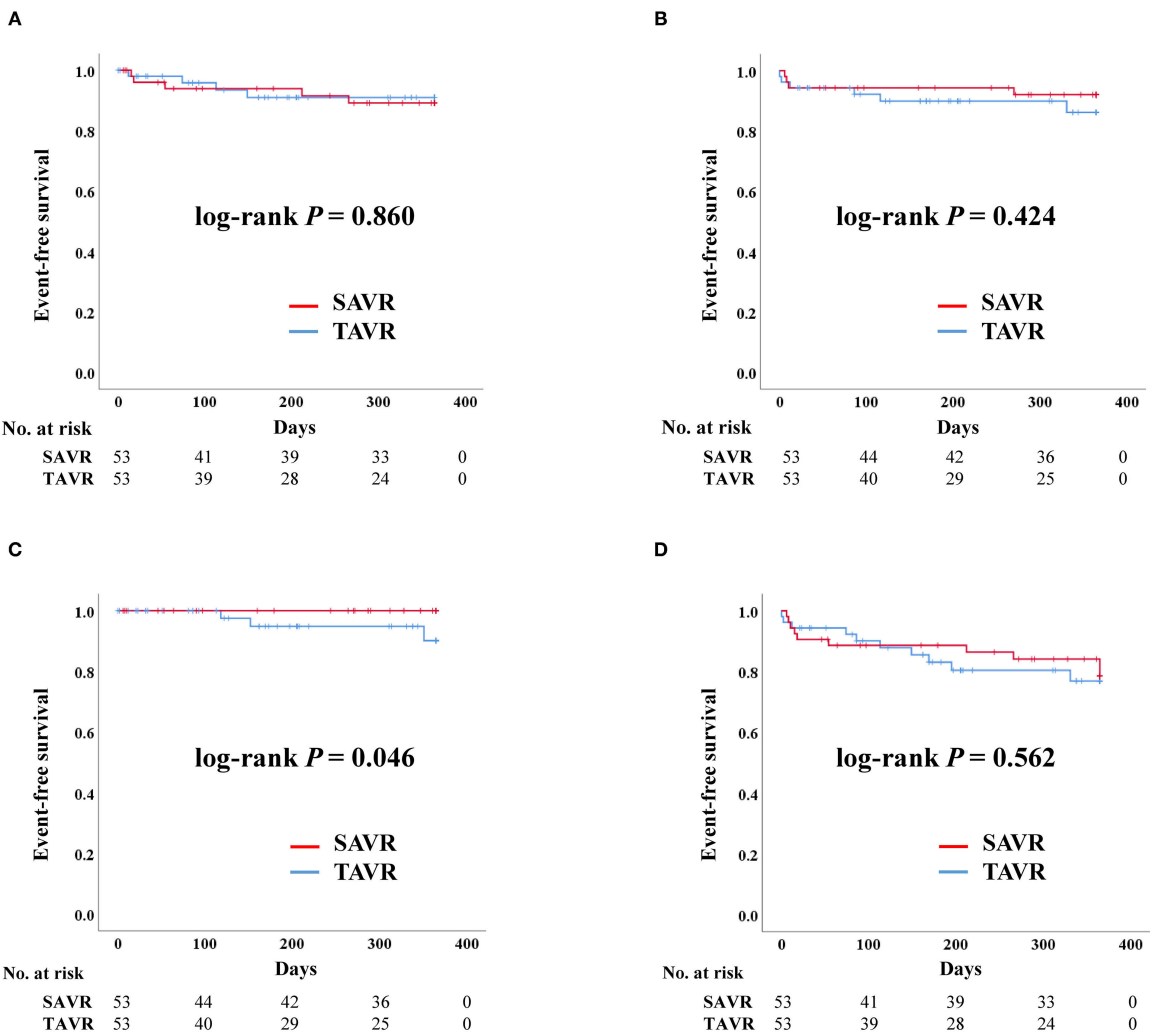
Clinical outcomes of surgical aortic replacement (SAVR) and transcatheter aortic valve replacement (TAVR) after propensity score matching. Composite outcome: composition of admission due to heart failure, stroke, or death. **(A)** Admission due to heart failure. **(B)** Death. **(C)** Stroke. **(D)** Composite outcome.

The rates of admission due to heart failure, stroke, death, and permanent pacemaker implantation were similar between the two groups before PS-matching. The composite outcomes were also comparable between the two groups (Table 3). These results were similar even after PS-matching. The Kaplan–Meier curve for event rates during the 1 year follow-up demonstrated that the rate of stroke was significantly higher in the TAVR than in the SAVR group. Otherwise, there were no significant differences in terms of admission due to heart failure, death, and composite outcome (Figure 1). Twelve patients in the SAVR group underwent AVR with a mechanical aortic valve. After PS-matching, only two patients with mechanical AVR were included in the analysis. These patients received warfarin after SAVR. All adverse clinical outcomes occurred in patients who underwent tissue AVR.

**Table 3:** Clinical event rates for 1 year follow-up.

	**Before propensity score matching**			**After propensity score matching**		
	**TAVR**	**SAVR**	***P*-value**	**TAVR**	**SAVR**	***P*-value**
	**(*n* = 21^†^/79^‡^)**	**(*n* = 12^§^/109^∥^)**		**(*n* = 21^†^/32^‡^)**	**(*n* = (2^§^/51^∥^)**	
Admission due to heart failure (%)	1^†^/4^‡^ (5.0)	8^∥^ (6.6)	0.685	4^‡^ (7.5)	5^∥^ (9.4)	0.860
Stroke	1^†^/4^‡^ (5.0)	3^∥^ (2.5)	0.436	1^†^/2^‡^ (5.7)	0 (0)	0.350
Ischemic	1^†^/3^‡^ (4.0)	1^∥^ (1.0)		1^†^/2^‡^ (5.7)	0 (0)	
Hemorrhagic	0 (0)	2^∥^ (1.7)		0 (0)	0 (0)	
TIA	1^‡^ (1.0)	0 (0)		0 (0)	0 (0)	
Death	1^†^/8^‡^ (9.0)	6^∥^ (5.0)	0.212	1^†^/5^‡^ (11.3)	4^∥^ (7.5)	0.429
PPM	1^†^/12^‡^ (13.0)	10^∥^ (8.3)	0.459	1^†^/6^‡^ (13.2)	4^∥^ (7.5)	0.270
Composite outcome^*^	3^†^/12^‡^ (15.0)	17^∥^ (14.0)	0.524	2^†^/8^‡^ (18.9)	10^∥^ (18.9)	0.564

*TAVR, Transcatheter aortic valve implantation; SAVR, Superficial aortic valve replacement; TIA, Transient ischemic accident; PPM, Permanent pacemaker*.

* *Composition of admission due to heart failure, stroke, or death;*

† *balloon expandable aortic valve;*

‡ *self-expandable aortic valve;*

§ *mechanical aortic valve;*

∥ *tissue aortic valve*.

#### Impact of AF History in TAVR

**Figure 2:**
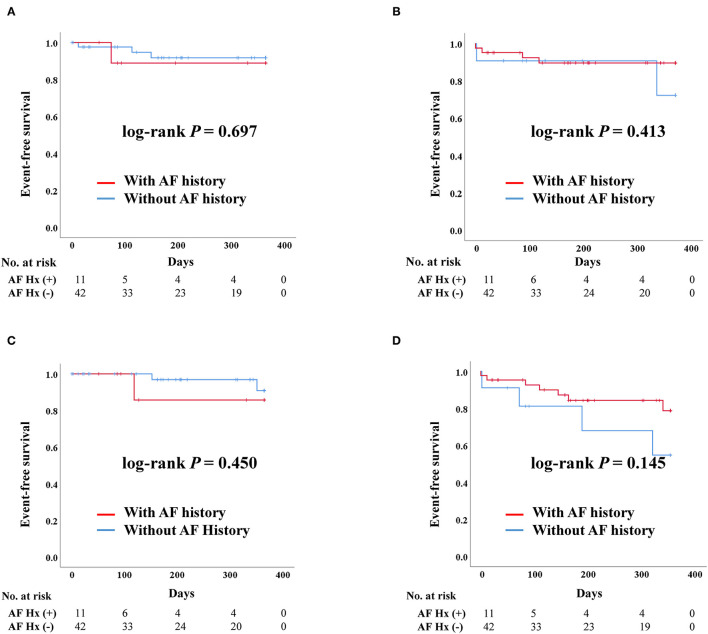
Clinical outcomes of transcatheter aortic valve replacement (TAVR) according to previous history of atrial fibrillation after propensity score matching. AF, Atrial fibrillation; Hx, History; Composite outcome, Composition of admission due to heart failure, stroke, or death. **(A)** Admission due to heart failure. **(B)** Death. **(C)** Stroke. **(D)** Composite outcome.

After PS-matching, the Kaplan–Meier curve demonstrated no significant difference in the rates of admission due to heart failure, death, stroke, and composite outcome, regardless of AF history. The occurrence of stroke tended to be higher in patients with a history of AF but there was no significant difference (Figure 2).

#### Impact of AF History in SAVR

**Figure 3:**
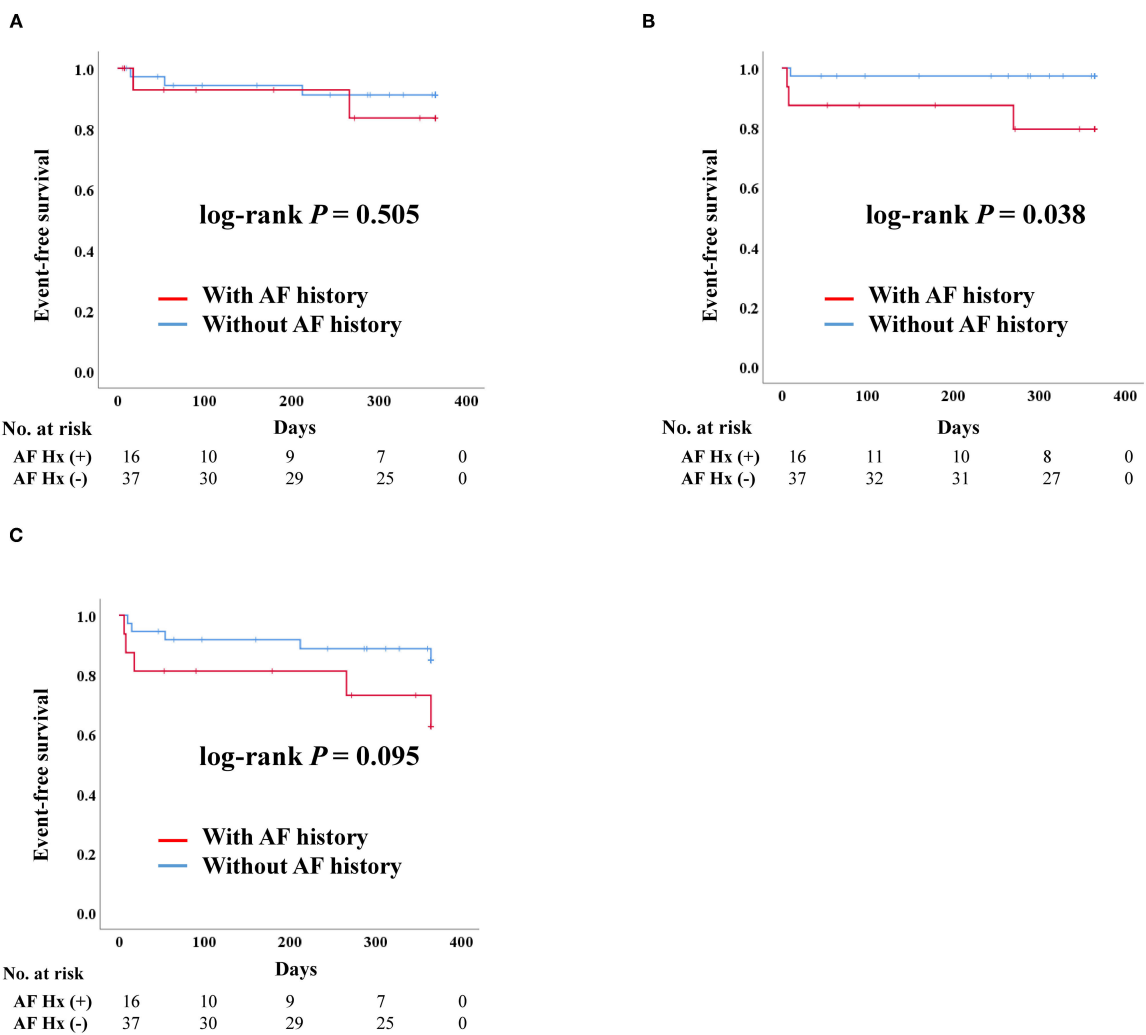
Clinical outcomes of surgical aortic replacement (SAVR) according to previous history of atrial fibrillation after propensity score matching. AF, Atrial fibrillation; Hx, History; Composite outcome, Composition of admission due to heart failure, stroke, or death. **(A)** Admission due to heart failure. **(B)** Death. **(C)** Composite outcome.

For patients who underwent SAVR, there was no significant difference in admission due to heart failure according to AF history. Death was more frequent in patients with AF than in those without a history of AF (log-rank *P* = 0.038). However, there were no stroke events in the SAVR group after PS-matching. The composite outcome was worse in patients with a history of AF, although there was no statistically significant difference (Figure 3).

#### Impact of Post-AVR AF in TAVR

**Figure 4:**
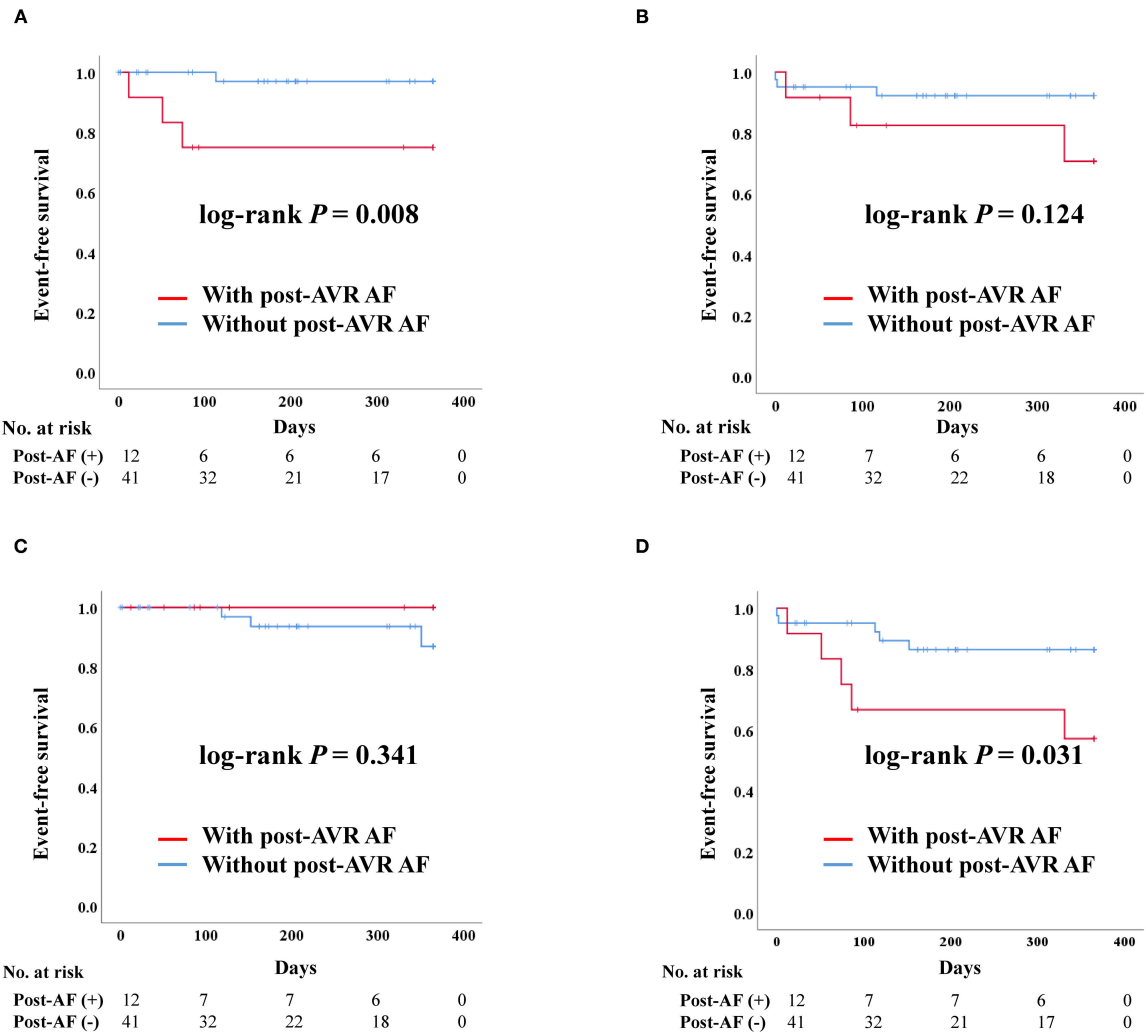
Clinical outcomes of transcatheter aortic valve replacement (TAVR) according to post-TAVR atrial fibrillation after propensity score matching. Post-AVR AF, Post-transcatheter aortic valve replacement atrial fibrillation; Composite outcome, Composition of admission due to heart failure, stroke, or death. **(A)** Admission due to heart failure. **(B)** Death. **(C)** Stroke. **(D)** Composite outcome.

After PS-matching in the TAVR group, admission due to heart failure was significantly higher in patients with post-AVR AF than those without (log-rank *P* = 0.008) (Figure 4). Death and stroke development were not significantly different between the two groups. Moreover, the composite outcome was better in patients without post-AVR AF (log-rank *P* = 0.031).

#### Impact of Post-AVR AF in SAVR

**Figure 5:**
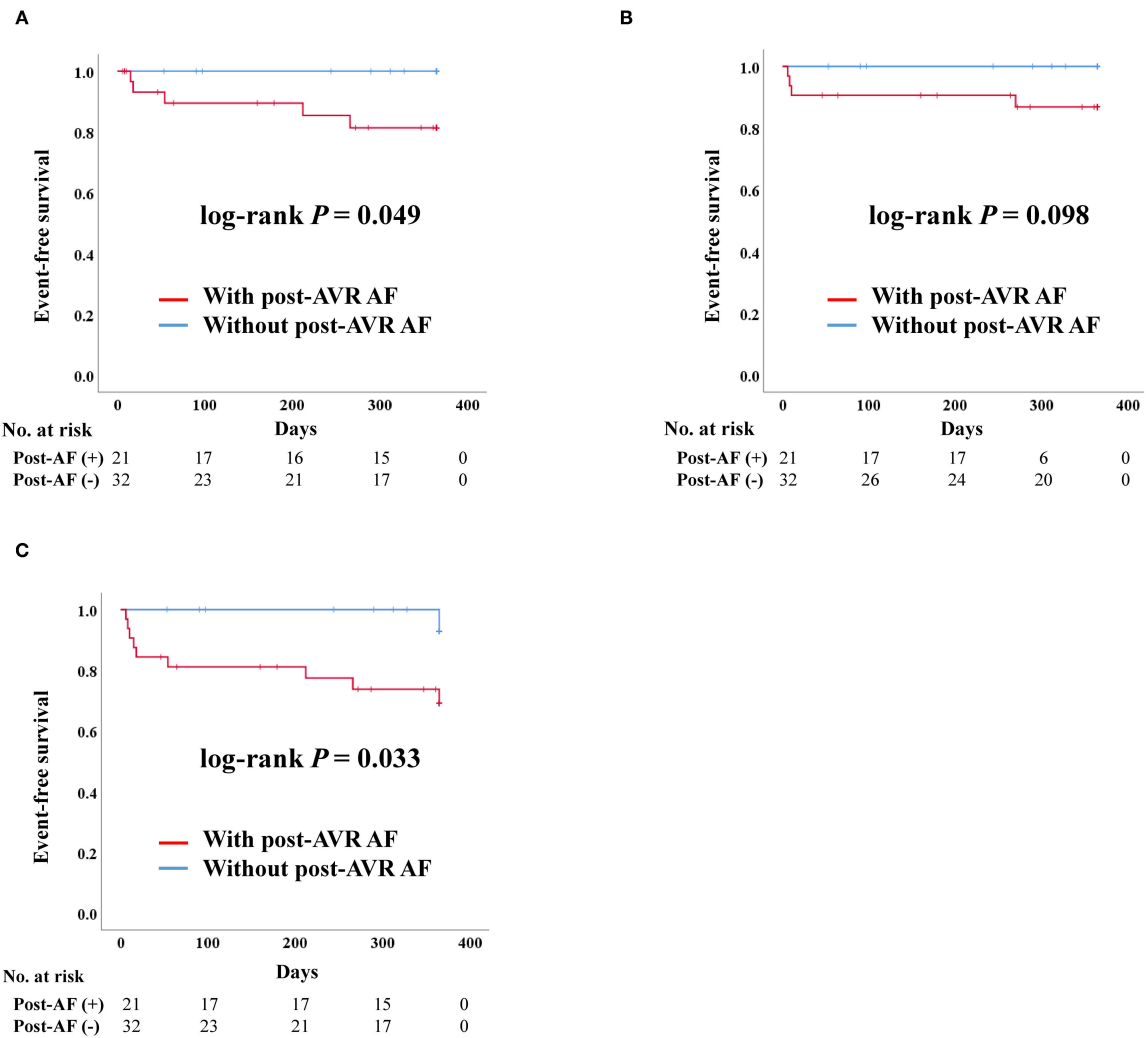
Clinical outcomes of surgical aortic valve replacement (SAVR) according to post-SAVR atrial fibrillation after propensity score matching. Post-AVR AF, Post-surgical aortic replacement atrial fibrillation; Composite outcome, Composition of admission due to heart failure, stroke, or death. **(A)** Admission due to heart failure. **(B)** Death. **(C)** Composite outcome.

After PS-matching in the SAVR group, admission due to heart failure and composite outcome was significantly worse in patients who developed post-AVR AF (Figure 5). There were no stroke events in the SAVR group after PS-matching. However, four deaths occurred, all of which were in the post-AVR AF group, although no statistical differences were observed in the log-rank analysis.

#### Risk Factors for Clinical Outcome After PS-Matching: TAVR vs. SAVR

In the univariate analysis using the Cox proportional model, post-AVR AF (hazard ratio [HR] = 3.58; confidence interval [CI] = 1.03–12.45; *P* = 0.044) and a previous history of myocardial infarction (HR = 4.98; CI = 1.39–17.78; *P* = 0.013) were associated with a worse composite outcome in the TAVR group. Conversely, no significant clinical factors were associated with worse outcomes in the SAVR group. In multivariate analysis, post-AVR AF (HR = 5.52; CI = 1.44–21.13; *P* = *0.013*, HR = 20.13; CI = 1.78–228.43; *P* = *0.015*) was associated with an adverse event for composite outcome. Additionally, a history of myocardial infarction (HR = 7.84; CI = 1.97–31.25; *P* = *0.004*, HR = 14.82; CI = 2.18–100.75; *P* = *0.006*) was also an independent risk factor for worse composite outcome in both TAVR and SAVR groups (Table 4).

**Table 4:** Risk factors of worse composite outcome in patients with TAVR and SAVR after PS-matching.

	**TAVR**						**SAVR**					
	**Unadjusted**			**Adjusted**			**Unadjusted**			**Adjusted**		
	**HR**	**95% CI**	***P*-value**	**HR**	**95% CI**	***P*-value**	**HR**	**95% CI**	***P*-value**	**HR**	**95% CI**	***P*-value**
Age	1.09	0.99–1.19	0.070				1.05	0.93–1.19	0.402			
Sex	0.53	0.14–2.03	0.351				0.94	0.26–3.34	0.924			
Hypertension	3.51	0.45–22.71	0.234				0.75	0.22–2.61	0.656			
DM	0.66	0.17–2.55	0.547				1.79	0.50–6.35	0.369			
Heart failure	1.33	0.28–6.28	0.720				2.05	0.43–9.66	0.366			
Previous MI	4.98	1.39–17.78	0.013	7.84	1.97–31.25	0.004	2.92	0.62–13.79	0.176	14.82	2.18–100.75	0.006
Old CVA	1.42	0.37–5.49	0.614				1.80	0.38–8.56	0.460			
CKD/ESRD	1.77	0.38–8.36	0.469				0.81	0.10–6.38	0.839			
AF history	3.00	0.84–10.68	0.090				2.75	0.80–9.52	0.110			
Post-AVR AF	3.58	1.03–12.45	0.044	5.52	1.44–21.13	0.013	6.88	0.87–54.37	0.067	20.13	1.78–228.43	0.015

*TAVR, Transcatheter aortic valve implantation; SAVR, Superficial aortic valve replacement; PS, propensity score; MI, Myocardial infarction; DM, Diabetes mellitus; CVA, Cerebrovascular accident; CKD, Chronic kidney disease; ESRD, End-stage renal disease; Post-AVR, Post-aortic valve replacement; HR, Hazard ratio; CI, Confidence interval*.

#### Comparison of Clinical Outcomes in Patients With Post-AVR AF: TAVR vs. SAVR

There was no significant difference in admission due to heart failure, death, or composite outcome between TAVR and SAVR group in patients with post-AVR AF after PS-matching (Supplementary Figure 1).

## Discussion

In the present study, we compared the incidence of AF between TAVR and SAVR after PS-matching, and analyzed the clinical outcomes according to the postoperative AF occurrence. Our results demonstrated that the incidence of post-AVR AF, especially newly detected AF, was significantly higher in the SAVR group before and after PS-matching (Table 2). Moreover, our data showed that AF had a worse impact on both TAVR and SAVR outcomes after PS-matching.

The incidence of AF in our study was consistent with that reported in previous studies. The incidence of newly detected AF in the TAVR group before and after PS-matching was 6 and 7.5%, respectively, while in the SAVR group, it was 40.5 and 35.6%, respectively. Previous studies have reported that the incidence of newly detected AF in TAVR ranges from 7.2 to 11.7% (3, 4, 12) and that the incidence of postoperative AF after cardiac surgery ranges from 10 to 65% (10, 11). In the recent New York state inpatient database validation cohort, the incidence of AF was 14.1% in patients undergoing TAVR and 30.6% in patients undergoing SAVR. Compared to our data, the incidence of AF in TAVR was higher, but, the incidence of AF in SAVR was less (13). The exact mechanism of AF development after TAVR has not yet been elucidated clearly. Although several mechanisms have been suggested for its pathophysiology, much of the knowledge is dependent on the mechanism of postoperative AF occurrence (14). Maesen et al. suggested that the factors facilitating AF can be divided into acute and chronic factors. Acute factors are directly related to procedures such as adrenergic stimulation, and chronic factors are associated with atrial remodeling, such as left atrium enlargement (15, 16). These factors may increase the risk of re-entry and ectopic activity, which may ultimately induce AF after cardiac surgery or TAVR (14). Urena et al. suggested that patients with severe AS develop AF because AF and AS share many common risk factors, such as age and hypertension. Moreover, they reported that one-third of the patients with AS were already affected by AF, which was more frequent than that of the TAVR group and less than that of the SAVR group in our study population (17).

The majority of postoperative AF is known to be terminated spontaneously within 4–6 weeks (10). Our results also demonstrated that most postoperative AF was terminated within 1 month (89.4%, 17 of 19). However, there are little data on how long newly detected AF persists after TAVR. Patients with AF who underwent TAVR at our center usually had AF for <3 months, except for one patient, in whom AF persisted for more than 3 months despite having a newly detected AF. Among the patients with newly detected AF (*n* = 6) in the TAVR group, AF was terminated within 1 month in three patients, within 3 months in two patients. Even though post-AVR AF was terminated relatively early in both the TAVR and SAVR groups, the data showed that AF still had a worse impact on clinical outcomes. Moreover, we believe that the AF was simply triggered by the procedure or surgery, and that the AF substrate had already existed. Therefore, the deleterious effects of AF would continue.

In one subgroup analysis of the PARTNER 3 trial, TAVR had a significantly lower frequency of post-AVR AF than did SAVR (18). This was similar to the present study's findings. Particularly, the authors divided AF into early and late postoperative AF. Only late postoperative AF, defined as AF occurring after hospital discharge up to 1 year, was associated with worse clinical outcomes regardless of treatment modalities. Although we did not categorize AF in detail, our results showed that post-AVR AF had a worse impact on clinical outcomes, which was consistent with that study. Therefore, the present study may provide evidence of real-world data pertaining to patients with various risk factors, which may be comparable to the subgroup analysis of well-designed randomized trials results.

Another subgroup analysis of the PARTNER 3 trial showed that low-risk patients with preexisting AF had worse clinical outcomes (19). The authors defined the outcomes as death, stroke, rehospitalization, and a composite of all these outcomes. Only the composite outcome was significantly higher in patients with pre-existing AF regardless of the treatment modalities, which was mainly driven by rehospitalization. However, when the patients were classified into SAVR and TAVR groups, AF did not significantly change the outcomes, although the absolute number of adverse outcomes was greater in the preexisting AF group. The present study also did not reveal significant differences in clinical outcomes according to AF history. However, considering the small sample size, the tendency of composite clinical outcome was similar to that reported in the recent study. Meanwhile, Zhang et al. suggested that preexisting AF is not an independent predictor of outcomes in severe AS (20). The authors assumed that the concomitant cardiac abnormalities may play a role in worse clinical outcomes. Therefore, whether preexisting AF has a substantially bad impact on the clinical outcome would require clarification through additional studies.

Usually, permanent pacemaker implantation rates are more frequent in TAVR than in SAVR. Interestingly, the rate of permanent pacemaker implantation was only 5.6% in the Korean data and 17% on average in other meta-analyses (21, 22). Yu et al. suggested that this was due to adequate device selection, meticulous procedures, high threshold for pacemaker implantation, relatively longer hospital stays, and low rate of pre-existing conduction disturbance (6). At our center, 13% of TAVR patients received a permanent pacemaker within 1 month, which was more frequent than that in the SAVR group, but the difference was not statistically significant.

The TAVR group had a higher incidence of stroke. In the present study, after PS-matching, there were no stroke events in the SAVR group, but there were three events in the TAVR group. Despite the significant difference, the data should be interpreted with caution because of the small sample size and lower event rates. We assumed that the occurrence of stroke in the TAVR group might have been attributable to guidewire and catheter manipulation through the aortic arch or calcified aortic valves. As TAVR is used in degenerative disease, many atherosclerotic changes in the aorta are likely to exist. Therefore, guidewire and catheter manipulation may induce devastating events, such as stroke. This finding is consistent with those of previous trials (23). However, a recent study showed that the stroke rate discrepancies between TAVR and SAVR dissipated 5 years after the procedure (24). However, the mechanism was unclear, and further research is required to assess it precisely.

Some data have previously demonstrated a comparison between TAVR and SAVR. Moreover, some data suggests that AF has a worse impact on clinical outcomes regardless of the AVR method. In this context, the novelty of our study is the investigation of the AF incidence after PS-matching and the comparison of the difference in the impact of AF on the clinical outcomes in each group. Our data showed that AF had worse impact on clinical outcomes in both groups, similar to the results of previous studies, and revealed a far higher incidence of AF in the SAVR group. This finding suggests that if the probability of AF occurrence is high in some patients with severe AS due to old age, hypertension, or chronic kidney disease, TAVR may be a favorable choice because of lower incidence of post-AVR AF.

AF-sustaining duration is relatively longer in cases of non-paroxysmal AF. Thus, non-paroxysmal AF may be considered to affect clinical outcomes. A research revealed that different AF types may impact clinical outcomes after TAVR. Shaul et al. suggested that histories of non-paroxysmal AF were associated with the risk of stroke or death. Conversely, paroxysmal AF did not significantly differ from sinus rhythm (25). They assumed that short AF episodes may be relative to the other major comorbidities; therefore, it would only have a minor effect on the overall clinical outcomes. In our study, a total of 27 patients had a history of AF after PS-matching. Paroxysmal AF (TAVR, *n* = 5, SAVR, *n* = 10) was identified in 15 patients, which was not sufficient for statistical analyses.

### Limitations

The present study had several limitations. First, the study population was enrolled from a single center in Korea. Therefore, it is not representative of all patients with severe AS. However, it is likely to reflect the effectiveness of TAVR or SAVR in Asian patients. Second, the sample size was relatively small, and discrepancies in the clinical outcomes between groups were smaller than those reported in previous studies, which revealed no statistical differences in some clinical outcomes. For example, there was no significant difference in the rate of permanent pacemaker implantations between the SAVR and TAVR groups. Third, this was a retrospective observational study, and the baseline characteristics were heterogeneous between the TAVR and SAVR groups. We conducted PS-matching to homogenize the baseline characteristics. However, as the differences in characteristics, such as age and previous myocardial infarction, were substantial, a large portion of the patients were excluded as a result.

#### Future Directions

Large-scale, multicenter, and prospective studies would be beneficial in overcoming the abovementioned limitations.

## Conclusion

TAVR and SAVR had similar clinical outcomes in patients with severe symptomatic AS, and post-AVR AF had a worse impact on both the TAVR and SAVR groups compared with that on patients without AF. Therefore, post-AVR AF could be considered a predictor of the outcomes of AVR. TAVR may be a more favorable treatment option than SAVR for patients with severe symptomatic AS who are at high-risk for AF-development or who have a history of AF because the occurrence of AF was less frequent in the TAVR group.

## Data Availability Statement

The raw data supporting the conclusions of this article will be made available by the authors, without undue reservation.

## Ethics Statement

The studies involving human participants were reviewed and approved by Ethics Committee of Chonnam National University Hospital, Gwangju, South Korea. The ethics committee waived the requirement of written informed consent for participation.

## Author Contributions

HKJ conducted the conception, investigation, and formal analysis and wrote the first draft of the manuscript. NY contributed to the design, literature search, experimental studies, data acquisition, data analysis, manuscript preparation, manuscript editing, manuscript review, approval of the final version of the manuscript, and agreement of all aspects of the work. All authors participated in the conception, investigation, manuscript review, approval of the final version of the manuscript, agreement of all aspects of the work, provided constructive criticism and comments, and read and approved the final manuscript.

## Funding

This study was supported by grants from the Chonnam National University Hospital Research Institute of Clinical Medicine (BCRI21011) and Korean Heart Rhythm Society (2020-01).

## Conflict of Interest

The authors declare that the research was conducted in the absence of any commercial or financial relationships that could be construed as a potential conflict of interest.

## Publisher's Note

All claims expressed in this article are solely those of the authors and do not necessarily represent those of their affiliated organizations, or those of the publisher, the editors and the reviewers. Any product that may be evaluated in this article, or claim that may be made by its manufacturer, is not guaranteed or endorsed by the publisher.

## Supplementary Material

Supplementary Figure 1 Transcatheter aortic valve replacement (TAVR) vs. surgical aortic valve replacement (SAVR) in patients with post aortic valve replacement (AVR) atrial fibrillation (AF) after propensity score (PS)-matching. There was no significant difference in the occurrence of heart failure, death or composite outcome in patients with post-AVR AF between TAVR and SAVR groups after PS-matching. It was impossible to compare stroke rates because there was no stroke event in SAVR group.
