# Supplementary figures and images for: Post-operative Atrial Fibrillation Impacts on Outcomes in Transcatheter and Surgical Aortic Valve Replacement
Source: Front Cardiovasc Med. 2021 Nov 29;8:789548. doi: 10.3389/fcvm.2021.789548 (PMC8667320; doi:10.3389/fcvm.2021.789548)

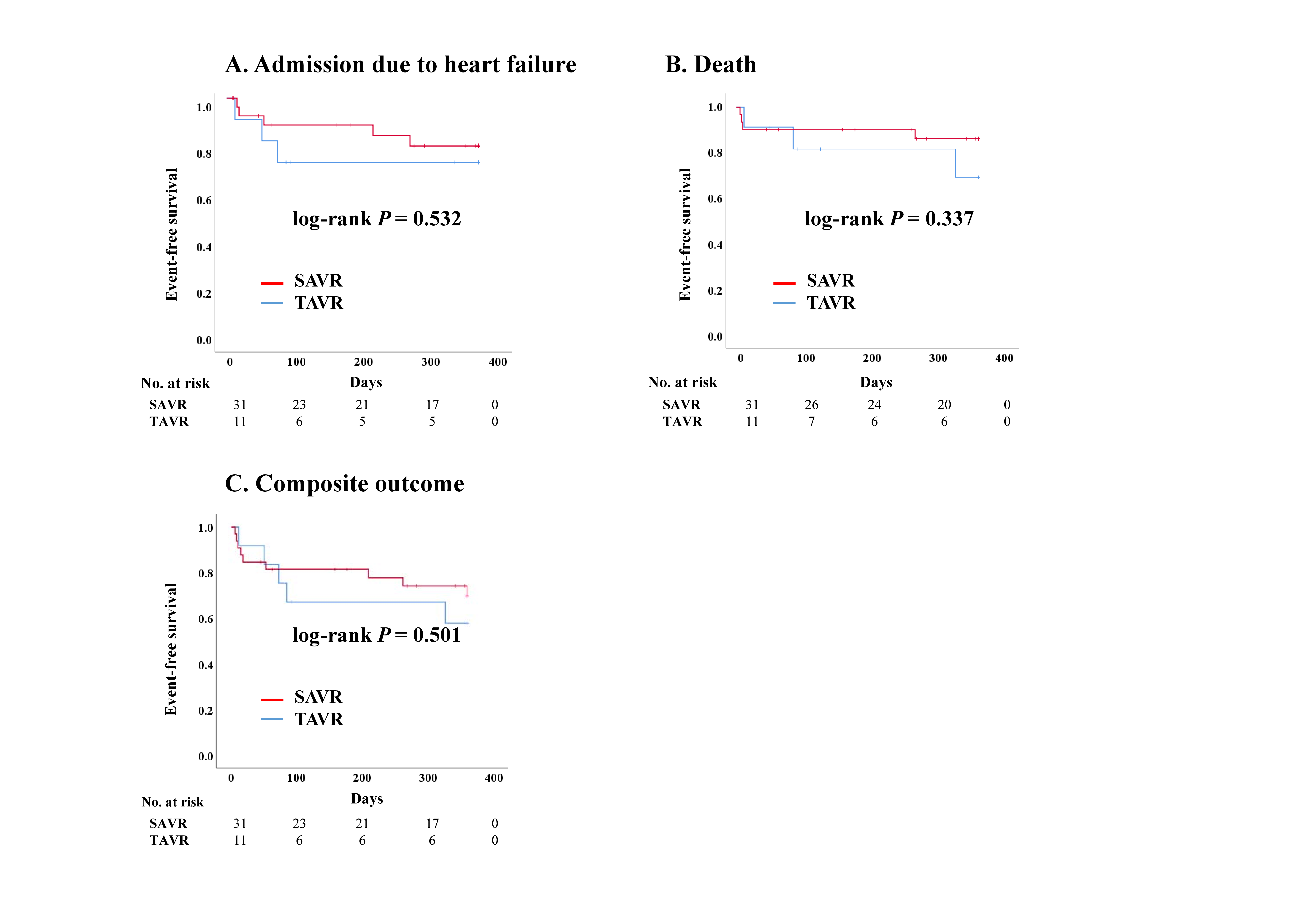

Supplement: Supplementary Figure 1 — Transcatheter aortic valve replacement (TAVR) vs. surgical aortic valve replacement (SAVR) in patients with post aortic valve replacement (AVR) atrial fibrillation (AF) after propensity score (PS)-matching. There was no significant difference in the occurrence of heart failure, death or composite outcome in patients with post-AVR AF between TAVR and SAVR groups after PS-matching. It was impossible to compare stroke rates because there was no stroke event in SAVR group. [file Image_1.TIFF]
